# Inflammatory-like presentation of CADASIL: a diagnostic challenge

**DOI:** 10.1186/1471-2377-12-78

**Published:** 2012-08-21

**Authors:** Nicolas Collongues, Nathalie Derache, Frédéric Blanc, Pierre Labauge, Jérôme de Seze, Gilles Defer

**Affiliations:** 1 Department of Neurology, Strasbourg University Hospital, 1, Avenue Molière, 67000, Strasbourg, France; 2 Department of Neurology, Caen University Hospital, Caen, 14000, France; 3 Department of Neurology, Montpellier University Hospital, Montpellier, 34000, France

**Keywords:** CADASIL, Multiple sclerosis, Leukoencephalopathy, Notch3, Cerebral vasculitis

## Abstract

**Background:**

CADASIL is an autosomal dominant genetic leukoencephalopathy linked to mutations in the *Notch3* gene. In rare cases, widespread brain lesions on T2 MRI mimicking multiple sclerosis are observed. From a national registry of 268 patients with adult-onset leukodystrophy, we identified two patients with an atypical presentation of CADASIL without co-occurrence of another systemic disease.

**Case presentations:**

Patient 1 experienced progressive gait disability and patient 2 relapsing optic neuritis and sensory-motor deficit in the leg. Both patients responded to corticotherapy and patient 2 was also responsive to glatiramer acetate. No oligoclonal bands were found in the CSF, and MRI showed myelitis and lesions with gadolinium enhancement in brain (patient 1) or incomplete CADASIL phenotype (patient 2).

**Conclusions:**

In rare cases, an inflammatory-like process can occur in CADASIL. In these patients, immunomodulatory treatments, including corticosteroids, could be effective.

## Background

CADASIL is an autosomal dominant vasculopathy that includes subcortical infarct and leukoencephalopathy. This disorder is linked to mutations in the *Notch3* gene, mostly located in exons 2-24 [1]. In rare cases, widespread brain lesions on T2 MRI mimicking multiple sclerosis (MS) are observed. To go further in the description of this specific presentation, patients were recruited through a national registry of 268 patients with adult-onset leukodystrophy fulfilling the following inclusion criteria: 1. age at onset above 16 years old; 2. symmetrical white matter involvement on the first MRI examination; 3. absence of autoimmune and infectious disorder tested by antinuclear, anti-neutrophil cytoplasmic, anti-phospholipid antibodies, VIH, lyme and syphilis blood tests, salivary gland biopsy, fundoscopy, CSF analysis and chest X-ray. Among them, we identified 23 patients with CADASIL, two of whom also presented atypical clinical and radiological signs mimicking MS. We describe these two cases and discuss the particularities of this inflammatory-like presentation of CADASIL.

## Case presentations

Patient 1. A 53-year-old woman with no previous personal or family medical history was admitted with a 6-month history of progressive gait disability and urinary urgency. Neurological assessment revealed dysesthesia in the feet and mild weakness in the left leg, brisk tendon reflexes in all four limbs and a bilateral Babinski sign. Neuropsychological examination showed a dysexecutive syndrome.

**Figure 1:**
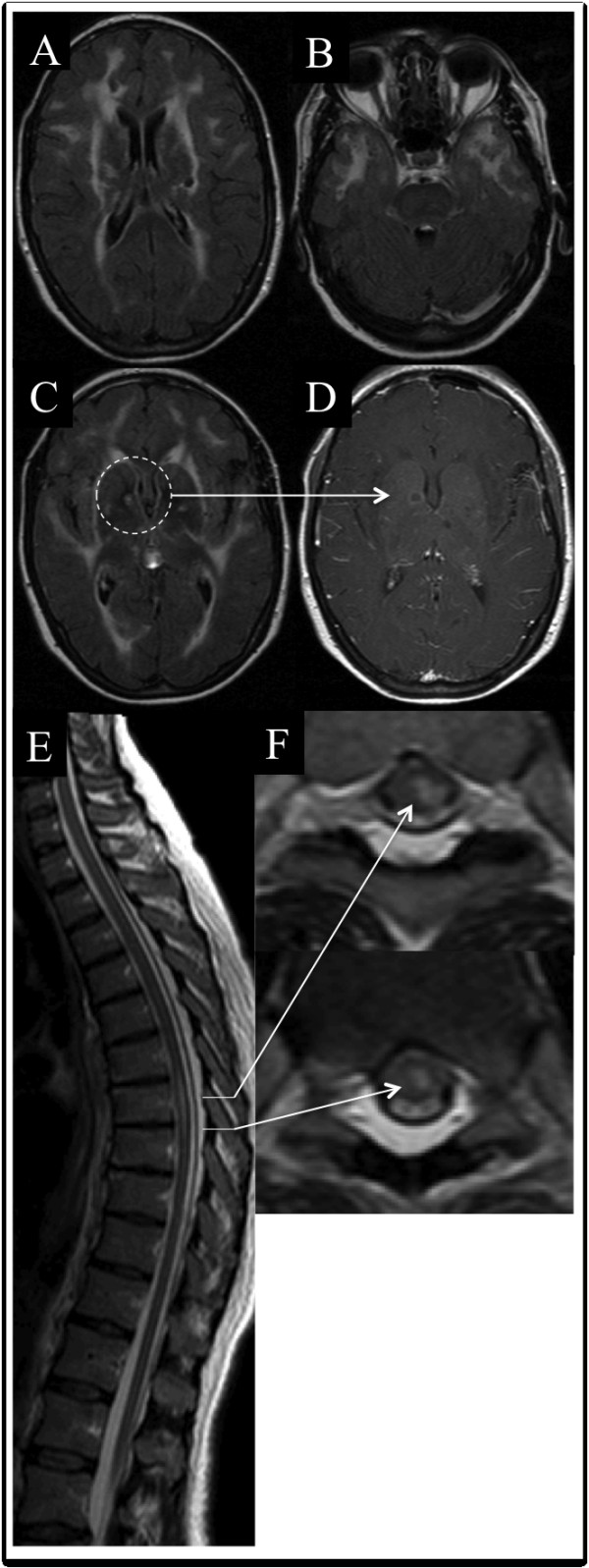
**Brain and spinal cord MRI of patient 1.** Flair sequence showing brain abnormalities typical of CADASIL, involving the external capsule, typical lacunar infarcts (**A**), and temporal lobes (**B**). Lesion in the right internal capsule (**C**) were enhanced by gadolinium injection on the T1-weighted image (**D**, arrow). A thoracic spinal cord lesion is noted on the longitudinal T2-weighted image (**E**), corresponding to partial myelitis in the transverse plane (**F**).

Blood samples and CSF analysis were normal, without any oligoclonal bands. Visual evoked potentials were normal. Spinal cord MRI showed thoracic hypersignal on T2-weighted images without gadolinium enhancement. Brain MRI showed extensive, bilateral, symmetrical periventricular and temporal hypersignal on T2-weighted images. One lesion, in the right internal capsule, was detectable on gadolinium-enhanced T1-weighted images (Figure 1).

A diagnosis of MS was suggested and treatment with intravenous corticosteroids was initiated, resulting in clinical improvement over the following few weeks. Owing to the extensive symmetrical lesions especially involving temporal lobes on brain MRI and despite the lack of family history of migraine, stroke or early-onset dementia, CADASIL was looked for. A missense mutation was found in exon 3 of *Notch3* at nucleotide 346 (c346C>T), resulting in a substitution of arginine for cysteine at codon 90. This mutation was not found in 200 Caucasian control individuals, thus confirming its pathogenic status.

Patient 2. A 46-year-old woman with a family history of MS (one affected cousin) experienced a first episode of optic neuritis followed by a sensory-motor deficit in the leg two years later.

Blood samples and CSF analysis were normal, without any oligoclonal bands. Visual evoked potential latencies were increased (128 ms on both sides). The initial brain MRI showed large T2-weighted lesions without gadolinium enhancement; the spinal cord MRI was normal.

**Figure 2:**
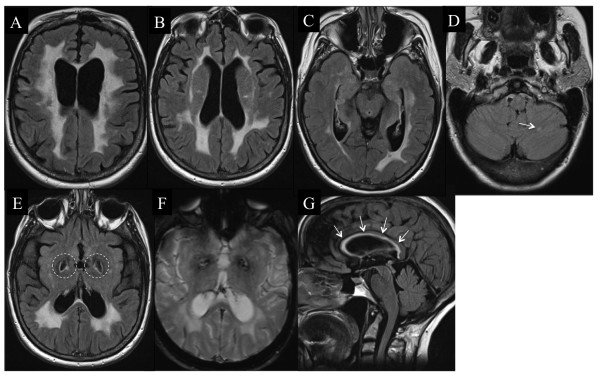
**Brain and spinal cord MRI of patient 2.** Flair sequence showing brain abnormalities typical of CADASIL (**A**) including the external capsule (**B**) but with numerous atypical findings for CADASIL: absence of temporal lobe involvement (**C**), cerebellar lesion in the left hemisphere (**D**, arrow), large bilateral cavities in the pallidum (**E**, dotted circles) without any microbleeds on T2*-weighted sequences (**F**), and a lesion in the corpus callosum, which has been rarely described in CADASIL (**G**, arrows).

During follow-up, she had experienced acute worsening once a year for 6 years before the first disease-modifying therapy and was responsive to methylprednisolone infusion. She was then diagnosed with MS and treated with glatiramer acetate. She experienced acute worsening of her condition on two occasions only, during the following 5 years of therapy. During the follow-up her brother and sister developed stroke-like episodes and were diagnosed with CADASIL. Owing to the aspect of brain MRI (Figure 2), the patient was also tested for CADASIL, and a missense mutation in exon 11 of *Notch3* was found. A C-to-T mutation at nucleotide 1750 (c.1750C>T) results in the substitution of arginine for cysteine at codon 558. This mutation was not found in 200 Caucasian control individuals, thus confirming its pathogenic status. After termination of the glatiramer acetate treatment acute worsening recurred once yearly. Glatiramer acetate was reintroduced and only one relapse occurred in the following 3 years. A neuropsychological assessment performed at the end of the follow-up showed hippocampal memory impairment, dysexecutive syndrome and mild depression.

## Conclusions

We have described two patients from different families with an inflammatory-like presentation of CADASIL. In patient 1, a cysteine had replaced arginine at codon 90 and in patient 2 arginine was substituted by cysteine at codon 558. These two mutations in *Notch3* gene, based on cysteine changes, correspond to the stereotyped mutations found in other reported patients with this diagnosis and are very likely pathogenic and a cause of CADASIL. The hallmark of these reports was the atypical clinical and MRI features in those patients that could rule out a diagnosis of CADASIL. The main unusual clinical findings were myelitis in patient 1 and optic neuritis in patient 2. Additional atypical findings were the lack of any history of migraine, corticosensitivity, and the absence of major disability over a long follow-up. Furthermore, unusual MRI findings for CADASIL were observed in patient 1: spinal cord lesion and gadolinium enhancement in the internal capsule. In patient 2, brain MRI did not show a typical CADASIL presentation, such as temporal involvement, lacunar infarct or microbleeds.

At first, whereas the diagnosis of CADASIL was genetically proven, in those patients, the occurrence of atypical presentation including myelitis and optic neuritis raised the question of an inflammatory process. Despite the lack of argument for an additional systemic autoimmune disorder, some findings such as a progressive spinal cord syndrome or relapsing optic neuritis, a possible therapeutic response to immunomodulators/corticosteroids, and MRI data, seemed to be in favor of MS. However, in those patients, the absence of oligoclonal bands and the aspect of extensive leukoencephalopathy on brain MRI with a microangiopathy aspect argued against that hypothesis. Furthermore, one should bear in mind that the improvement after anti-inflammatory treatment may have been overestimated because some degree of recovery from small ischemic lesions cannot be excluded and because the course of other inflammatory diseases of the CNS are often unpredictable.

An association of two diseases is also possible including CADASIL and MS: possibly a progressive form in patient 1 and a relapsing form in patient 2, fulfilling the MS diagnostic criteria with the limit that another diagnosis can be discussed (the no better explanation criteria). It is to note that, in the literature, no co-occurrence of CADASIL and MS has been found in any of 93 MS patients with a family history of MS [2]. Despite those results, we had learned that, from a physiopathogenic point of view, genetic or epigenetic events could play a role to modify the course of diseases, including notch protein and signalling pathway in CADASIL and MS [3], or mitochondrial cytopathies such as Leber’s disease or *POLG* mutations which have been suggested to promote MS [4,5].

Another hypothesis is that our patients had atypical CADASIL in which an inflammatory process occurred mimicking MS. In the literature, some of the above four findings evoking MS have, rarely, been associated with CADASIL, including a steroid-responsive spinal cord syndrome in one patient [6] and progressive disability in another patient [7]. The physiopathological mechanism invoked to explain those particularities in CADASIL remains unclear and involved chronic hypoperfusion for progressive disability [8] or T-cell modulation, as observed in animal models [9]. Increased vascular permeability of the blood–brain barrier could also be hypothesized because the accumulation of Notch-3 receptor ectodomain in the vascular wall is known to impair its structural and functional stability [10]. At last, some metabolism disorders may produce new epitopes like myelin or neuronal debris leading to non specific inflammatory reactions as observed in our patients.

The aim of this study was to sensitize clinicians to such atypical presentations of CADASIL and not to establish epidemiological data. Selection biases were inherent in the constitution of the CADASIL cohort, because some patients may not have been declared, while others may have been classified as MS-patients because mutations in the *Notch3* gene have not been researched. Whatsoever, the reported prevalence of CADASIL lies between 4 to 15 cases per 100000 [1], which therefore makes the co-existence of another rare event improbable.

In conclusion, inflammatory-like presentation can occur in CADASIL. This condition should be known to clinicians because it could represent a challenge to make the right diagnosis. In these patients, immunomodulatory treatments, including corticosteroids, could be effective and should be proposed in the absence of validated therapeutic for CADASIL.

## Competing interests

The authors declare that they have no competing interests.

## Authors’ contributions

NC coordinated the study under the supervision of PL, JdeS and GD. ND and FB enabled the recruitment of patients in each medical center. PL developed the leukodystrophy cohort of patient with an adult-onset. PL, JdeS and GD had an advisory board in the project. NC, ND, FB, PL, JdeS and GD carried out the analysis and interpretation of data. NC has drafted of the manuscript. All authors read and approved the final manuscript.

## Consent form

Detail has been removed from these case descriptions to ensure anonymity. Written informed consent was obtained from the patients for publication of these case reports and any accompanying images. A copy of the written consent is available for review by the Editor-in-Chief of this journal.

## Funding

None

## Pre-publication history

The pre-publication history for this paper can be accessed here:

http://www.biomedcentral.com/1471-2377/12/78/prepub
